# First chemo-enzymatic synthesis of the (*R*)-Taniguchi lactone and substrate profiles of CAMO and OTEMO, two new Baeyer–Villiger monooxygenases

**DOI:** 10.1007/s00706-016-1873-9

**Published:** 2016-12-21

**Authors:** Florian Rudroff, Michael J. Fink, Ramana Pydi, Uwe T. Bornscheuer, Marko D. Mihovilovic

**Affiliations:** 1 Institute of Applied Synthetic Chemistry, TU Wien, Getreidemarkt 9/163-OC, 1060 Vienna, Austria; 2 Department of Chemistry and Chemical Biology, Harvard University, 12 Oxford St, Cambridge, MA 02138 USA; 3 Department of Biotechnology and Enzyme Catalysis, Institute of Biochemistry, University of Greifswald, 17487 Greifswald, Germany

**Keywords:** Biotransformation, Baeyer–Villiger oxidation, Taniguchi lactone

## Abstract

**Abstract:**

This study investigates the substrate profile of cycloalkanone monooxygenase and 2-oxo-Δ^3^-4,5,5-trimethylcyclopentenylacetyl-coenzyme A monooxygenase, two recently discovered enzymes of the Baeyer–Villiger monooxygenase family, used as whole-cell biocatalysts. Biooxidations of a diverse set of ketones were performed on analytical scale: desymmetrization of substituted prochiral cyclobutanones and cyclohexanones, regiodivergent oxidation of terpenones and bicyclic ketones, as well as kinetic resolution of racemic cycloketones. We demonstrated the applicability of the title enzymes in the enantioselective synthesis of (*R*)-(−)-Taniguchi lactone, a building block for the preparation of various natural product analogs such as ent-quinine.

**Graphical abstract:**

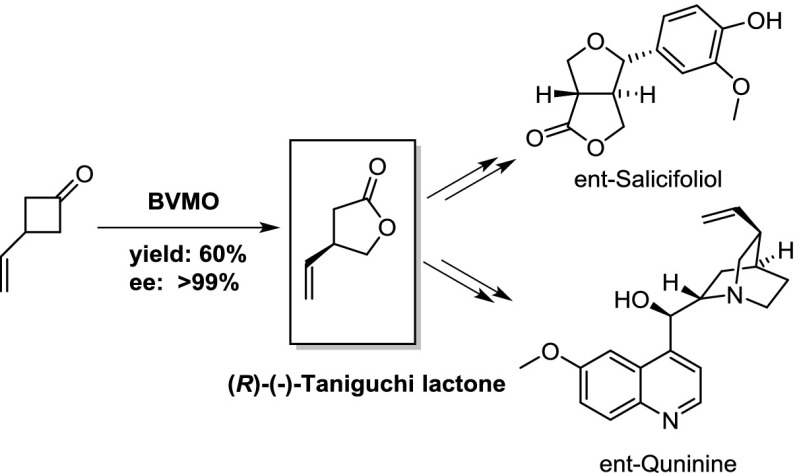

## Introduction

Baeyer–Villiger monooxygenases are well-known enzymes, promoting the oxidation of cyclic and linear ketones into the corresponding lactones or esters by activating molecular oxygen in water at ambient temperature. This enzyme family catalyzes the biological equivalent to the chemical Baeyer–Villiger oxidation, discovered by Adolf Bayer and Victor Villiger in 1899 [1], which requires peracids and rather harsh reaction conditions. Major drawbacks of the classic stoichiometric synthetic route are the poor tolerance of other functional groups (e.g., towards double bonds and heteroatoms), the need of potentially explosive reagents (e.g., *meta*-chloroperbenzoic acid), and the reduced possibility of chiral induction [2–10].

The resulting esters and, especially if obtained optically enriched, chiral lactones are promising building blocks for the synthesis of valuable intermediates of drugs and natural products [11]. During the last decades, applicability of BVMOs has been demonstrated in multiple accounts: desymmetrization of prochiral substrates, kinetic resolutions of racemic ketones, and regioselective transformations [12–19]. We had previously profiled the substrate scope of a range of BVMOs [20–24], allowing us to correlate their stereopreference with protein sequence [25]. This led to a significant clustering of BVMOs into subgroups, and eventually to the development of a decision guidance tool [24], enabling a pre-selection of best-performing biocatalysts for a particular substrate class.

**Fig. 1:**
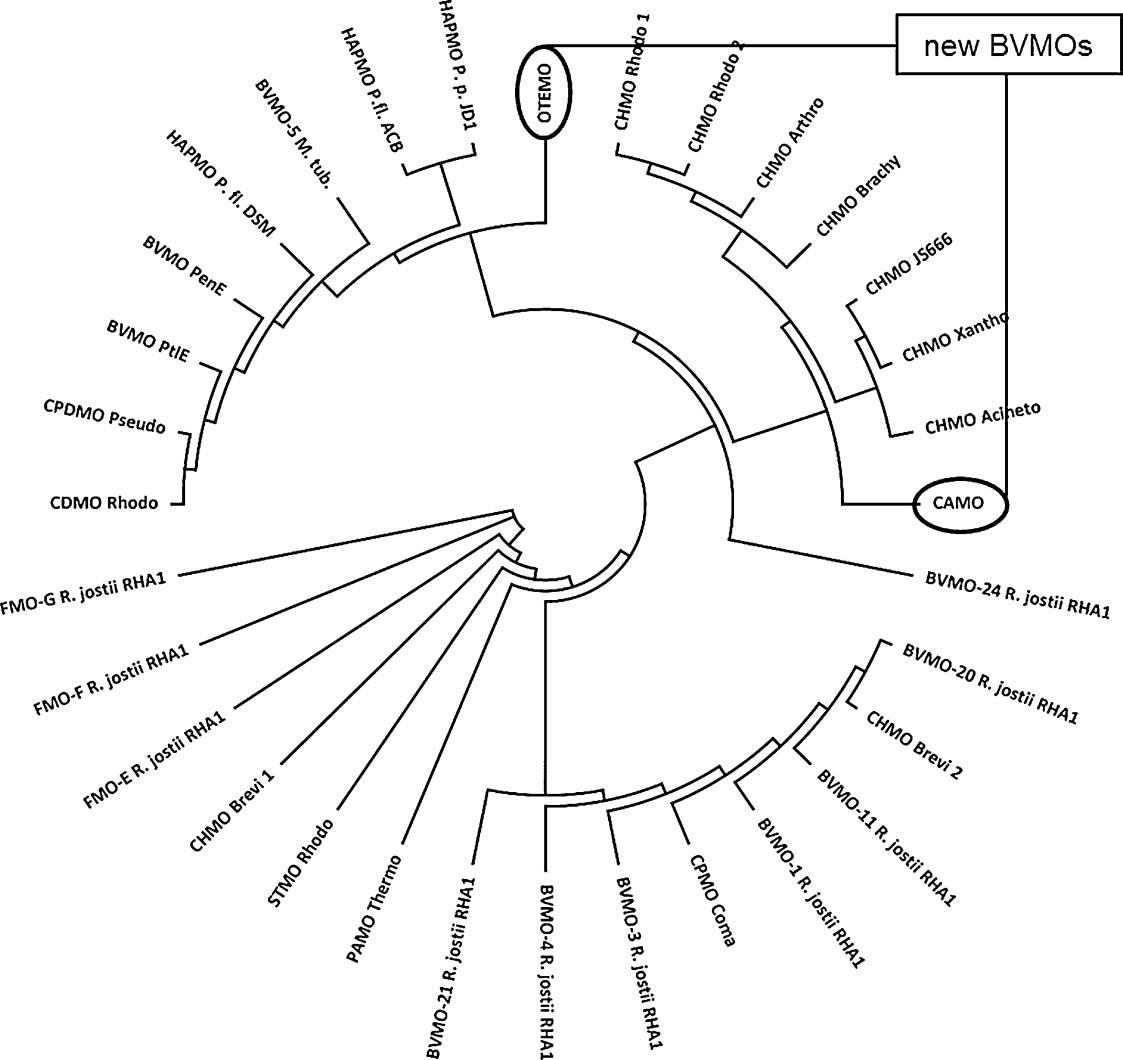
Phylogenetic tree of BVMOs. The sequences of the investigated enzymes CAMO and OTEMO are* highlighted*. CAMO belongs to the CHMO_Acineto_ subgroup, whereas OTEMO clustered separately from known groups of correlated sequence and stereopreference

Camphor monooxygenase (CAMO) from the ascomycete *Cylindrocarpon radicicola* ATCC 11011 was recently reported as the first recombinant BVMO originating from a eukaryotic organism [26]; it possesses a general activity for both cyclic and linear ketones. In contrast, recombinant 2-oxo-Δ^3^-4,5,5-trimethylcyclopentenylacetyl-coenzyme A monooxygenase (OTEMO) from *Pseudomonas putida* NCIMB 10007 showed activity for cyclic ketones, preferentially [27, 28]. Whenever new BVMOs are discovered, it is of particular interest to know their activity, catalytic performance, and selectivity in relation to previously characterized enzymes. The distinct positions of CAMO and OTEMO in the phylogenetic tree (Fig. 1) of BVMOs motivated our efforts of in-depth substrate profiling of these new biocatalysts.

Furthermore, we wished to demonstrate the applicability of both title enzymes by developing a new biocatalytic approach for the synthesis of optically pure (*R*)-(−)-Taniguchi lactone. The enantiomeric (*S*)-(+)lactone [29] has been used as a building block for the synthesis of various natural products, e.g., salicifoliol, threosyl-5′-deoxyphosphonic acid, and purine analogs [30], including the first stereoselective synthesis of quinine [31]. Several attempts succeeded to obtain the Taniguchi lactone in optically pure form via chiral resolution by chiral agents [32–34]. Synthetic accessibility of the (*R*)-(−)-Taniguchi lactone will provide a novel approach for the synthesis of, e.g., ent-quinine and ent-salicifoliol, respectively.

Here, we present a comprehensive substrate profile of two novel BVMOs (CAMO, OTEMO) and the first chemo-enzymatic synthesis of the (*R*)-(−)-Taniguchi lactone in enantiopure form via a desymmetrization step.

## Results and discussion

We investigated the sequence homology of CAMO and OTEMO. Their divergent positioning in the phylogenetic analysis (CAMO clustered with CHMO-type enzymes, whereas OTEMO branched off at an early point; Fig. 1) suggested a distinctly different substrate profile between the biocatalysts. To determine the catalyst performance a diverse array of substrates (e.g., 2- and 4-substituted cyclohexanones, 3-substituted cyclobutanones, and terpenones) was investigated. The experiments were conducted on analytical scale and the results obtained were compared and referenced to published values. The substrates were grouped into kinetic resolutions, regiodivergent oxidations, and desymmetrization reactions.

### Kinetic resolutions

We tested a set of α-substituted cyclohexanones, starting from small methyl **1a**, to allyl **2a**, and more bulky substituents, including phenyl **3a** and benzyl **4a** residues (Scheme 1). The enantiomeric ratio *E* was estimated using Sih’s equation.

**Figure:**
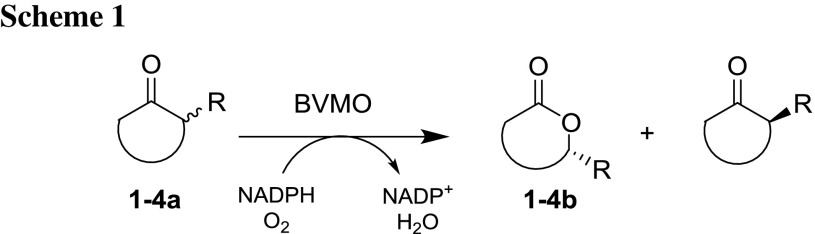

*E* = [ln(1 − eeS) − ln(1 + eeS/eeP)]/[ln(1 + eeS) − ln(1 + eeS/eeP)],

from ee of substrate (eeS) and ee of product (eeP) values, and compared to already published reference biotransformations (Table 1). Since CAMO is closely related in sequence to CHMO-type enzymes, we expected its stereopreference to align with the cluster; the hypothesis was found to be true with all tested compounds of the class. Also, OTEMO had the same sense of chiral induction. Nevertheless, except for the transformation of 2-phenylcyclohexanone with CAMO (*E* > 200), the title enzymes were only poorly selective (*E* = 3–69), when compared to the best reference results using CDMO or CHMOs.

**Table 1:** Kinetic resolutions of 2-substituted cyclohexanones

Substrate	*R*	Comp. no	CAMO			OTEMO		
			Conv (%)^a^	% ee S/% ee P	E^b^	Conv (%)^a^	% ee S/% ee P	*E* ^b^
	Me	**1**	56	86(−)/60(+)	11	33	23(−)/40 (+)	3
	Allyl	**2**	46	53(−)/88(−)	28	60	43(−)/50(−)	5
	Ph	**3**	48	96(−)/99(+)	>200	32	43(−)/95(+)	64
	Bn	**4**	54	62(−)/91(−)	41	58	73(−)/94(−)	69

Substrate	*R*	Comp. no	Reference biotransformation				
			Conv (%)^a^	% ee S/% ee P	E^b^	Biocatalyst	References
	Me	**1**	58	99(+)/92(−)	125	CDMO	[24]
	Allyl	**2**	50	99(−)/99(−)	>200	CHMO_Acineto_	[35]
	Ph	**3**	48	76(−)/99(+)	>200	CHMO_Xantho_	[36]
	Bn	**4**	47	91(−)/99(−)	>200	CDMO	[24]

^a^Relative conversion (Conv) of starting material and enantiomeric excess values determined by chiral phase GC; sign of optical rotation is given in parentheses and assigned on the basis of reference biotransformations; ee_S_ (substrate), ee_P_ (product)

^b^Enantiomeric ratio *E* was estimated using Sih’s equation

### Regiodivergent biotransformations

The second set of substrates was composed of fused bicyclic cyclobutanones and monocyclic terpenones to test the regioselectivity of CAMO and OTEMO. For these particular substrate classes the formation of two regioisomers is often observed: the “normal” lactone (*n*), based on the nucleophilicity-driven rearrangement, and the “abnormal” lactone (*abn*) governed by stereoelectronic effects (Scheme 2) [36].

**Figure:**
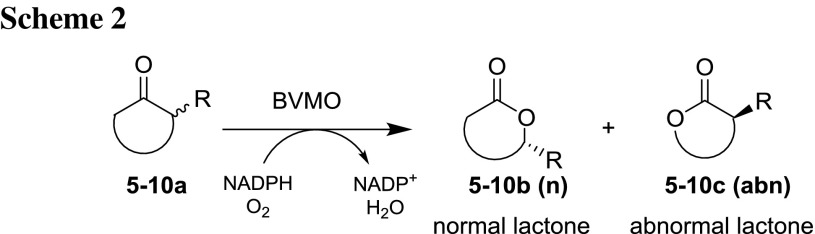

In general, fused bicyclic cyclobutanones are good substrates for BVMO-mediated biooxidations due to the highly favored alleviation of ring strain by insertion of an oxygen atom (Table 2). Ketone **5a** was fully converted by CAMO and gave an equal ratio of *n* and *abn* lactones **5b/c**. Enantioselectivity for the *n* lactone was good [ee 88% (−)] and excellent for the *abn* lactone [ee 99% (−)]. Again, CAMO gave comparable results to previously published data of CHMO-type BVMOs. In contrast, OTEMO gave **5b/c** in a 70/30 ratio of *n*/*abn* with moderate stereoselectivity for the *n* lactone [ee 46% (−)] and excellent optical purity for the *abn* lactone [ee 99% (−)]. Contrasting results were obtained for ketone **6a**: whereas CAMO gave a 70/30 *n*/*abn* ratio with poor [**6b**: ee 41% (+)] to excellent [**6c**: ee 98% (+)] enantioselectivities, OTEMO transformations yielded in a 1/1 ratio of *abn*/*n* lactone with perfect optical purity for both regioisomers. This result is the best obtained so far for this compound (**6a**). The last example of this substrate class (**7a**) was fully converted by both enzymes, with slight preference towards the *abn* lactone **7c**. Optical purities were moderate to poor for the *n* lactone **7b** and high for the *abn* lactone **7c** for both enzymes.

**Table 2:** Regiodivergent Baeyer–Villiger oxidations of fused and racemic (*cis*) bicyclic cyclobutanones

Substrate	Comp. no	CAMO		OTEMO		Reference biotransformation			
		% Conv^a^/ ratio^b^	% ee^c^ *n*/*abn*	% Conv^a^/ ratio^b^	% ee^c^ *n*/*abn*	% Conv^a^/ ratio^b^	% ee^c^ *n*/*abn*	Biocatalyst	References
	**5**	+++ 53:47	88(−)/99(−)	+++ 70:30	46(−)/99(−)	+++ 97:03	rac./99(+)	CPMO	[37]
						+++ 51:49	95(−)/99(−)	CHMO_Acineto_	[25]
	**6**	+++ 70:30	41(+)/98(+)	+++ 50:50	97(+)/99(+)	+++ 87:13	14(+)/99(+)	CPMO_Coma_	[37]
						+++ 70:30	44(+)/99(+)	CHMO_Acineto_	[37]
	**7**	+++ 68:32	46(+)/99(+)	+++ 89:11	20(+)/95(+)	+++ 75:25	33(−)/56(−)	CPMO_Coma_	[37]
						+++ 97:3	12(+)/88(+)	CHMO_Acineto_	[37]

*rac.* racemic

^a^Relative conversion (Conv) of starting material determined by chiral phase GC after 24 h: +++>90%, ++50–90%, +<50%

^b^Ratio of regioisomers (normal:abnormal)

^c^Enantiomeric excess values determined by chiral phase GC; sign of optical rotation is given in parenthesis and assigned on the basis of reference biotransformations

A second tier of substrates for regiodivergent reactions consisted of optically pure terpenones (**8**–**10a**). Previous studies revealed that CHMO family enzymes preferably gave *n* lactones, whereas CPMO-type enzymes formed *abn* lactones [10]. With those compounds, the preference of migration strongly depends on the absolute configuration of the starting material. We tested three different terpenones **8**–**10a** with CAMO and OTEMO (Table 3). The former showed poor regioselectivity; in contrast OTEMO was highly selective in the oxidation of substrates **9a** and **10a**, yielding the n lactones **9**, **10b** exclusively. These results demonstrated that neither CAMO nor OTEMO behaved like a classical CHMO- or CPMO-type BVMO.

**Table 3:** Regiodivergent transformations of optically pure terpenones

Substrate	Comp. no	CAMO^c^	OTEMO^c^	Reference biotransformation^c^		
		% Conv^a^/ ratio^b^	% Conv^a^/ ratio^b^	% Conv^a^/ ratio^b^	Biocatalyst	References
	**8**	+++ 53:47	+++ 11:89	+++ 0:100	CHMO_Acineto_	[22]
				++ 51/49	CHMO_Brevi1_	[22]
	**9**	+++ 70:30	+++ 100:0	+++ 100:0	CHMO_Acineto_	[22]
				+++ 0:100	CPMO_Coma_	[22]
	**10**	+++ 68:32	+++ 100:0	+++ 100:0	CHMO_Acineto_	[22]

^a^Relative conversion (Conv) of starting material determined by chiral phase GC after 24 h: +++>90%, ++50–90%, +<50%

^b^Ratio of regioisomers (normal:abnormal)

^c^Enantiomeric excess >99%

### Desymmetrization reactions

A series of 11 prochiral ketones was biooxidized by CAMO and OTEMO in desymmetrization reactions (Scheme 3; Table 4). All 3-substituted cyclobutanones **11**–**15a** were fully converted to the lactones **11**–**15b**. 3-Vinylcyclobutanone **11a** was transformed with perfect stereoselectivity by both tested BVMOs (both giving (−)-**11b** with 99% ee).

**Figure:**
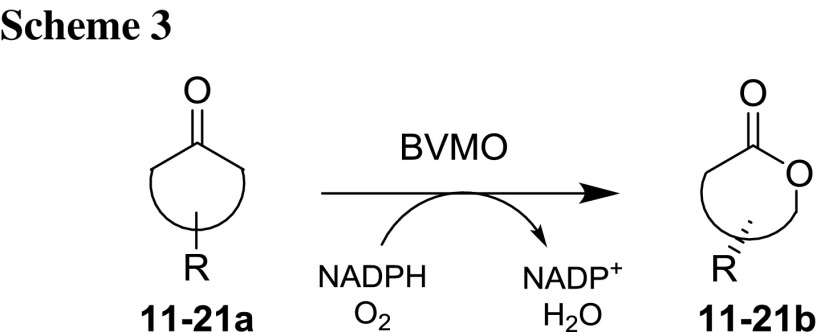

**Table 4:** Desymmetrizations of substituted prochiral cycloketones **11**–**21a**

Structure	*R*	Comp. no	CAMO		OTEMO		Reference biotransformation			
			Conv (%)^a^	ee (%)^b^	Conv (%)^a^	ee (%)^b^	Conv (%)^a^	ee (%)^b^	Biocatalyst	References
	3-Vinyl	**11**	100	99(−)	100	99(−)	n.a.	n.a.	n.a.	This work
	Bn	**12**	99	97(+)	98	11(−)	+++	44(+)	HAPMO	[43]
							+++	93(−)	CHMO_Brevi1_	
	3-(3-MeOBn)	**13**	100	94(−)	100	82(−)	+++	63(−)	CPMO_Coma_	[43]
	3-(4-MeOBn)	**14**	95	95(+)	100	64(+)	+++	24(+)	CPMO_Coma_	[43]
	3-(3,4,5-tri-MeOBn)	**15**	100	92(+)	87	63(+)	+++	79(+)	CHMO_Brevi1_	[43]
	Me	**16**	100	99(−)	100	58(+)	+++	99(−)	CDMO	[24]
							+++	64(+)	CPMO_Coma_	[44]
	OH	**17**	100	>99(+)	n.a.	n.a.	+++	99(−)	CHMO_Xantho_	[24]
							+++	44(+)	CHMO_Brevi2_	
	*t*Bu	**18**	98	77(−)	100	98(+)	+++	99(−)	CPDMO	[18]
	COOEt	**19**	99	95(−)	57	96(+)	+++	99(−)	CPDMO	[18]
							+++	64(+)	CPMO_Coma_	[18]
	H, H	**20**	100	97(−)	100	96(−)	+++	99(−)	CDMO	[24]
	H, OH (*trans*)	**21**	100	96(+)	100	94(+)	+++	96(+)	CHMO_Acineto_	[45]

*n.a*. not applicable

^a^Relative conversion (Conv) of starting material determined by chiral phase GC after 24 h: +++>90%, ++50–90%, +<50%

^b^Enantiomeric excess values determined by chiral phase GC; sign of optical rotation is given in parenthesis and assigned on the basis of reference biotransformations

CAMO’s stereopreference for cyclobutanones **12**–**14a** was divergent from other CHMO-type BVMOs; it produces the enantiomers usually obtained by CPMO-type transformations, but with much higher selectivity (Table 4). Lactones **12b** and **14b** were thus obtained with 97% (+) and 95% (+) ee. The optical purity of lactones **13b**, **15b** was much higher than previously published values [**13b** = 94% (−) vs 63% (−) and **15b** = 92% (+) vs 73% (+)]. These lactones are important intermediates for the synthesis of tricyclic benzomorphan analogs, (+)-harzialactone A, chiral tricyclic amines [38–40], and for the synthesis of lignans such as enterolactone, hinokinin, arctigenin [41], the synthesis of optically pure gosmin A and schizandrin [42]. In all cases, OTEMO had the same stereopreference as CAMO, but was less stereoselective.

Both enzymes fully converted all tested 4-substituted cyclohexanones (substrates **16**–**21a**). Remarkably, lactone **17b** was formed with perfect enantiomeric purity [ee 99%, (+)] using CAMO, thus surpassing the best known result. Both optical antipodes of lactone **18b** could be obtained by CAMO and OTEMO, respectively: whereas CAMO gave the levorotatory isomer with 77% ee, OTEMO yielded in the antipodal product with 98% ee. A similar trend was observed for substrate **19a**, where CAMO produced (−)-**19b** with 95% ee, and OTEMO gave (+)-**19b** with 96% ee. The latter result again exceeded the best previously obtained value with wild-type BVMOs.

Substrates **20a** and **21a** were transformed with very good enantioselectivity by both enzymes, as commonly found with other BVMOs.

### Chemo-enzymatic synthesis of (*R*)-(−)-Taniguchi lactone

After identification of CAMO and OTEMO as excellent catalysts for the enantioselective synthesis of lactone **11b**, we investigated a new chemo-enzymatic route to Taniguchi lactone **11b**, starting from readily available 1,3-butadiene. This lactone is a valuable chiral building block for the synthesis of several natural products (Scheme 4).

**Figure:**
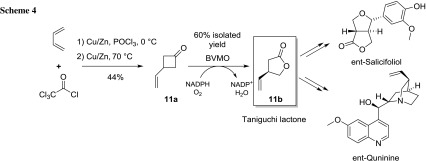

We performed a [2 + 2] cycloaddition using dichloroketone, generated in situ from trichloroacetyl chloride in the presence of activated zinc [46]. Subsequent reduction of the dichloroketone led to 3-Vinylcyclobutanone **11a** in 44% over two steps. We then screened a library of cycloketone-converting BVMOs, composed of 13 wild-type enzymes, plus mutants of CHMO_Acineto_ and CPMO_Coma_, that had been generated in previous studies [47, 48].

Most wild-type BVMOs gave the desired Taniguchi lactone with excellent conversion and optical purity (ee > 95%), exclusively with (*R*)-configuration (data not shown). All mutants of CHMO_Acineto_ that where designed for enantiodivergence towards cyclobutanones and cyclohexanones, showed a reduced stereospecificity, and no inversion of the selectivity was observed with any biocatalyst. A similar trend was seen using the variants of CPMO_Coma_. We then performed biotransformation on a preparative scale (50 mg) with CAMO (57% isolated yield) and OTEMO (60% isolated yield) to confirm the results obtained in the screening reactions on the analytical scale. Overall, we were able to synthesize enantiomerically pure Taniguchi lactone **11b** in 26% yield over three steps having ee 99% (−) optical purity by biocatalytic desymmetrization.

## Conclusion

In this work, we reported the substrate scope and compared the performance of CAMO and OTEMO as whole-cell biocatalysts overexpressed in *E. coli.* We investigated commercially available and in-house synthesized racemic 2-substituted cyclohexanones, fused bicyclic cyclobutanones, optically pure terpenones, prochiral cyclobutanones and cyclohexanones, all either serving as model substrates or intermediates in the synthesis of natural compounds. The biocatalysts gave remarkable results in the desymmetrization of cyclobutanones and -hexanones, in some cases improving the enantioselectivity significantly over prior best values. The other reaction classes were generally not converted with a performance equal to reference BVMOs.

A biocatalytic route for the synthesis of optically pure (*R*)-(−)-Taniguchi lactone was established, starting from 1,3-butadiene via biooxidation of 3-Vinylcyclobutanone with CAMO and OTEMO as recombinant whole-cell catalysts. This synthesis is an interesting alternative approach to known preparations of the target lactone.

## Experimental

All purchased reagents were used without further purification. All solvents were distilled prior to use. Substrates and reference compounds for substrate acceptance screening were synthesized according to literature-known protocols. Silica gel 60 was used for column chromatography and progress of the reactions was monitored by thin layer chromatography (TLC). The analysis and identification of all molecules were conducted using chiral phase GC (BGB 173 and BGB 175 Column: 30 m × 0.25 mm ID, 0.25 μm film). NMR spectra were recorded on a Bruker AC 200 (200 MHz) spectrometer. Specific rotation was determined using an Anton Paar Polarimeter MCP500. LB medium was not buffered and used without pH correction; ampicillin and kanamycin concentrations were set to 100 mg/cm^3^ (LB_amp_ and LB_kan_). Cloning, expression and characterization of CAMO [26] and OTEMO [27, 28] is described elsewhere.

### Substrate acceptance screening reactions

A baffled Erlenmeyer flask was charged with 3 cm^3^ LB_amp_ medium, inoculated with a bacterial single colony from an agar plate, and incubated at 37 °C and 200 rpm in an orbital shaker overnight. LB_amp_ medium (10 cm^3^) was inoculated with 1% (v/v) of the overnight culture and incubated for 1–2 h under the same conditions until an optical density of 0.2–0.6 was reached. Then, 2 cm^3^ of fermentation medium was charged to 12-well plates and IPTG (0.2 mM) was added. Substrates were added as 0.8 M solutions in 1,4-dioxane to a final concentration of 4 mM. The plates were sealed with adhesive film and incubated at 24 °C and 200 rpm in an orbital shaker for up to 24 h. Analytical samples were prepared by extraction of 0.5 cm^3^ of fermentation mixture with 1.0 cm^3^ of EtOAc (supplemented with 1 mM methyl benzoate as internal standard) after centrifugation to separate the biomass.

#### 3-Vinylcyclobutanone (**11a**)

1,3-Butadiene (1 g, 18.5 mmol, 49 cm^3^ of a 15 wt% solution of 1,3-butadiene in hexane) was added to freshly prepared and dry Cu/Zn couple [49] (3.5 g, 52 mmol) at 0 °C under Ar atmosphere. A mixture of 9.5 g trichloroacetyl chloride (52 mmol) and 7.8 g POCl_3_ (50 mmol) was added dropwise at 0 °C under argon within 2–4 h. The mixture was stirred overnight at r.t., zinc salts were removed by filtration through a pad of Celite; the filtrate was diluted with 150 cm^3^ of diethyl ether and carefully diluted with 100 cm^3^ of cold water. The organic phase was separated and washed with aq. NaHCO_3_ (4 × 50 cm^3^), then with 100 cm^3^ brine and dried over anhydrous Na_2_SO_4_. The solvent was removed in vacuo and the residue was used in the next step without further purification.

The crude residue (2.1 g) was dissolved in 10 cm^3^ acetic acid and added dropwise to a suspension of zinc in 30 cm^3^ acetic acid within 1 h, followed by heating to 70–80 °C for another hour. The reaction mixture was cooled to r.t., filtered through a pad of Celite, diluted with 100 cm^3^ water, and extracted with dichloromethane (4 × 50 cm^3^). The combined organic phases were washed with saturated aq. NaHCO_3_ (4 × 50 cm^3^), then with 100 cm^3^ brine, dried over anhydrous Na_2_SO_4_, and the solvent was removed in vacuo to obtain the desired product **11a** as a yellow oil. Yield: 44% (782 mg) over two steps; ^1^H NMR (200 MHz, CDCl_3_): *δ* = 6.02 (ddd, 1H), 5.01–5.36 (m, 2H), 3.39 (s, 1H), 2.89–3.36 (m, 4H) ppm; ^13^C NMR (50 MHz, CDCl_3_): *δ* = 206.7, 140.2, 114.6, 52.6, 26.9 ppm.

#### *rac*-4-Vinyldihydrofuran-2(3H)-one (Taniguchi lactone) (**11b**)

2-Buten-1,4-diol (6 g, 68 mmol), 22 g triethyl orthoacetate (136 mmol), and 2.25 g *p*-hydroquinone (20.4 mmol) were stirred at 120 °C. Generated ethanol was removed using a Dean-Stark apparatus. After the condensation of ethanol had ceased, the temperature was increased to 150 °C for 24 h. The crude residue was isolated via distillation under reduced pressure (60–70 °C, 36 mbar) and the product was obtained by column chromatography *rac*-**11b** as a yellow oil to serve as reference material for chiral analytics. Yield: 42% (3.35 g); ^1^H NMR (200 MHz, CDCl_3_): *δ* = 5.76 (m, 1H), 5.07–5.27 (m, 2H), 4.42 (m, 1H), 4.00 (m, 1H), 3.21 (m, 1H), 2.65 (m, 1H), 2.36 (m, 1H) ppm; ^13^C NMR (50 MHz, CDCl_3_): *δ* = 176.5, 135.7, 117.4, 72.1, 39.7, 34.1 ppm.

#### (*R*)-4-Vinyldihydrofuran-2(3H)-one (Taniguchi lactone) (**11b**)

An overnight culture with LB_kan_ medium (3 cm^3^), inoculated with a bacterial single colony from an agar plate and incubated at 37 °C and 200 rpm in an orbital shaker was prepared. A baffled Erlenmeyer (500 cm^3^) flask was charged with 100 cm^3^ of LB_kan_ medium and inoculated with 1% (v/v) of the overnight culture and incubated for 1–2 h under the same conditions until an optical density of 0.2–0.6 was reached. IPTG (0.2 mM) and 50 mg ketone **11a** (0.52 mmol, dissolved in 200 mm^3^ of 1,4-dioxane) was added. The reaction flask was incubated at 24 °C and 200 rpm in an orbital shaker until full conversion was determined via GC analysis (18–24 h). After completion of the reaction, the reaction mixture was centrifuged (12000 x g, 15 min, 4 °C). The supernatant was extracted with EtOAc (5 × 40 cm^3^), and the pooled organic phases were dried over anhydrous Na_2_SO_4_, and concentrated. The product was purified by column chromatography using light petroleum and EtOAc. Yields: 57% (33 mg) for CAMO and 60% (35 mg) for OTEMO. ^1^H NMR and ^13^C NMR were identical to the racemic Taniguchi lactone [32]. [*α*]_D_^25^ **=** −7.0 (*c* = 1 in CHCl_3_), 99% ee.

## References

[1] Baeyer A, Villiger V (1899). Chem Berichte.

[2] Leisch H, Morley K, Lau PC (2011). Chem Rev.

[3] Mihovilovic MD, Rudroff F, Groetzl B (2004). Curr Org Chem.

[4] Ito K (2012) In: Asymmetric Baeyer-Villiger oxidation. Elsevier B.V., Amsterdam, p 1

[5] Michelin RA, Sgarbossa P, Scarso A, Strukul G (2010). Coord Chem Rev.

[6] Sugiishi T, Matsugi M, Hamamoto H, Amii H (2015). RSC Adv.

[7] Uyanik M, Ishihara K (2013). ACS Catal.

[8] Xu S, Wang Z, Zhang X, Zhang X, Ding K (2008). Angew Chem Int Ed.

[9] Watanabe A, Uchida T, Ito K, Katsuki T (2002). Tetrahedron Lett.

[10] Bolm C, Luong TKK, Schlingloff G (1997) Synlett 1151

[11] Mihovilovic MD (2006). Curr Org Chem.

[12] Kamerbeek NM, Janssen DB, van Berkel WJH, Fraaije MW (2003). Adv Synth Catal.

[13] Bucko M, Gemeiner P, Schenkmayerova A, Krajcovic T, Rudroff F, Mihovilovic MD (2016). Appl Microbiol Biotechnol.

[14] Muschiol J, Peters C, Oberleitner N, Mihovilovic MD, Bornscheuer UT, Rudroff F (2015). Chem Commun.

[15] Rudroff F, Fink MJ, Mihovilovic MD, Goswami A, Stewart JD (2016). Miscellaneous key non-C–C bond forming enzyme reactions. Organic synthesis using biocatalysis.

[16] Leipold F, Rudroff F, Mihovilovic MD, Bornscheuer UT (2013). Tetrahedron Asymmetry.

[17] Fink MJ, Fischer TC, Rudroff F, Dudek H, Fraaije MW, Mihovilovic MD (2011). J Mol Catal B Enzym.

[18] Fink MJ, Rudroff F, Mihovilovic MD (2011). Bioorg Med Chem Lett.

[19] Oberleitner N, Peters C, Muschiol J, Kadow M, Sass S, Bayer T, Schaaf P, Iqbal N, Rudroff F, Mihovilovic MD, Bornscheuer UT (2013). ChemCatChem.

[20] Mihovilovic MD, Müller B, Schulze A, Stanetty P, Kayser MM (2003) Eur J Org Chem 2243

[21] Mihovilovic MD, Rudroff F, Müller B, Stanetty P (2003). Bioorg Med Chem Lett.

[22] Cernuchova P, Mihovilovic MD (2007). Org Biomol Chem.

[23] Fink MJ, Fischer TC, Rudroff F, Dudek H, Fraaije MW, Mihovilovic MD (2011). J Mol Catal B Enzym.

[24] Fink MJ, Rial DV, Kapitanova P, Lengar A, Rehdorf J, Cheng Q, Rudroff F, Mihovilovic MD (2012). Adv Synth Catal.

[25] Mihovilovic MD, Rudroff F, Grötzl B, Kapitan P, Snajdrova R, Rydz J, Mach R (2005). Angew Chem Int Ed.

[26] Leipold F, Wardenga R, Bornscheuer UT (2012). Appl Microbiol Biotechnol.

[27] Kadow M, Loschinski K, Sass S, Schmidt M, Bornscheuer UT (2012). Appl Microbiol Biotechnol.

[28] Leisch H, Shi R, Grosse S, Morley K, Bergeron H, Cygler M, Iwaki H, Hasegawa Y, Lau PC (2012). Appl Environ Microbiol.

[29] Kondo K, Mori F (1974). Chem Lett.

[30] Lian Jin Liu EK, Hong Joon Hee (2012). Nucleosides Nucleotides Nucleic Acids.

[31] Stork G, Niu D, Fujimoto A, Koft ER, Balkovec JM, Tata JR, Dake GR (2001). J Am Chem Soc.

[32] von Kieseritzky F, Wang Y, Axelson M (2014). Org Process Res Dev.

[33] Ishibashi F, Taniguchi E (1998). Phytochem.

[34] Ishibashi F, Taniguchi E (1988). Bull Chem Soc Jpn.

[35] Rial DV, Cernuchova P, van Beilen JB, Mihovilovic MD (2008). J Mol Catal B Enzym.

[36] Noyori R, Sato T, Kobayashi H (1983). Bull Chem Soc Jpn.

[37] Mihovilovic MD, Kapitan P, Kapitanova P (2008). ChemSusChem.

[38] Kotkar SP, Suryavanshi GS, Sudalai A (2007). Tetrahedron Asymmetry.

[39] Ketterer C, Wünsch B (2012). Eur J Org Chem.

[40] Ketterer C, Grimme S, Weckert E, Wünsch B (2006). Tetrahedron Asymmetry.

[41] Bode JW, Doyle MP, Protopopova MN, Zhou Q-L (1996). J Org Chem.

[42] Tanaka M, Mukaiyama C, Mitsuhashi H, Wakamatsu T (1992). Tetrahedron Lett.

[43] Rudroff F, Rydz J, Ogink FH, Fink M, Mihovilovic MD (2007). Adv Synth Catal.

[44] Wang S, Kayser MM, Iwaki H, Lau PCK (2003). J Mol Catal B Enzym.

[45] Mihovilovic MD, Rudroff F, Grötzl B, Stanetty P (2005) Eur J Org Chem 809

[46] Dehmlow EV, Kinnius J, Buchholz M, Hannemann D (2000). J Prakt Chem Chem-Ztg.

[47] Mihovilovic MD, Rudroff F, Winninger A, Schneider T, Schulz F, Reetz MT (2006). Org Lett.

[48] Clouthier CM, Kayser MM, Reetz MT (2006). J Org Chem.

[49] Rudroff F, Rydz J, Ogink FH, Fink M, Mihovilovic MD (2007). Adv Synth Catal.

